# Editorial: Un/belonging identities: relating narratives of queer trauma

**DOI:** 10.3389/fsoc.2025.1691021

**Published:** 2025-10-14

**Authors:** Mark Vicars, Jonathan Glazzard, Liam Wrigley, Jordan Gonzalez

**Affiliations:** ^1^COABLIT & ISIIC, Victoria University, Melbourne, VIC, Australia; ^2^School of Education, University of Hull, Hull, United Kingdom; ^3^School of Social Policy and Society, University of Birmingham, Birmingham, United Kingdom; ^4^School of Education, St John's University, New York, NY, United States

**Keywords:** queer (LGBTQ), trauma, victimization, (un)belonging, crime, education, heteronormativity, hostile environment

## Introduction

In this Research Topic of *Frontiers in Sociology, Gender, Sex and Sexualities*, the materiality of complex trauma, as encountered and experienced by LGBTQIA+ individuals, is articulated across 16 papers that address cultural landscapes of queer citizenship and draw on how binary architectures of meaning get reproduced through the grammar of (hetero)cultural narratives. The growth of inequality, evidenced in the spread of LGBTQIA+ hate crimes, is rapidly becoming a dialectical re-engagement with the concepts and practices of inclusion and exclusion. Heteronormative practices of *being* and *belonging* habitually operate as a site of power and privilege that can make queer bodies feel “out of place”. The authors in this Research Topic draw upon a spectrum of onto-epistemic violences, linguistically performed and physically aimed at queer bodies with the aim of reproducing articulations of (dis)connectedness and discomfort. Such weaponization brings to the fore the haunting presence of traumatic (dis)affection as a form of world and self-making.

As a collective of queer academics, we recognize that we occupy a relatively privileged position which provides us with the opportunity to undertake research that, we hope, will advance social justice. However, trauma and discrimination are integrated into our daily lives. Although we are thriving, we continue to experience trauma. Queer trauma is multilayered and complex (Weststrate et al., 2024; Wrigley and Koumentaki). It is rooted in socially manufactured heterosexist and cisgendered discourses that regulate the way people think, speak, and act. Microaggressions, discrimination, and other forms of oppression are experienced both individually and collectively—they are pervasive. Oppressive social policies and attacks on LGBTQIA+ rights globally have created “queer battle fatigue” (Morris et al., 2022; Wozolek et al., 2015) and perpetuated stigma and shame. The HIV/AIDS crisis in the early 1980s, which positioned queer people as a contagion, is a powerful example of how wicked narratives are spread, resulting in collective trauma. Across the world, queer people have experienced a long history of individual and collective trauma due to being criminalized, mocked, silenced, tortured, and killed. For some, these appalling atrocities against LGBTQIA+ communities are unfortunately woven into the fabric of their daily lives (Coleman-Fountain, 2014). As researchers, we cannot stand by, watch this happen, and remain silent. We have a duty to use our voices to amplify those voices that have been silenced.

## Overview of the edited Research Topic

This edited volume brings together a diverse range of original research articles, conceptual analyses, perspectives, community case studies, and autoethnographic accounts that collectively interrogate queer trauma and un/belonging identities across cultural, educational, and social contexts. The Research Topic is deliberately transnational, drawing from a wide variety of geographical locations and cultural experiences, including India, Vietnam, South Africa, Norway, Australia, the Dominican Republic, the United States, and the United Kingdom, as well as transnational and diasporic communities. This breadth allows the volume to situate queer trauma not as a singular phenomenon but as one that is experienced, mediated, and resisted differently across local, national, and global contexts.

In *Interrogating global narratives of trans queerness. Well-being and agency? Or more stories of trans trauma?*, *Vicars and Milenkovic* situate contemporary anti-trans discourses within a psycho-social tapestry of collective trauma, foregrounding the urgent need for trauma-informed understandings of trans life worlds. *Criminalised, victimised or other? A reflexive engagement with Queer Criminology utilising a relational pedagogical approach* (Wrigley and Koumentaki) considers how queer criminology and relational pedagogy unsettle binary constructions of victimhood and criminalization, opening space for more complex understandings of trauma in queer lives. *Experiences of trans women who have undergone gender affirmation surgery: a constructivist grounded theory* (Dharsheni and Sivakami) examines the lived experiences of trans women in Chennai who have undergone both traditional and medicalized forms of gender affirmation surgery, revealing the entanglement of cultural practices, stigma, and resilience. Complementing this, *Life experiences leading to the choice of surgery—A qualitative study exploring reasons behind the choice of undergoing gender affirmative surgery* (Bjørnson and Sagbakken) offers a Norwegian perspective that underscores how socio-cultural norms and enacted stigma shape the decision-making processes of individuals pursuing gender-affirming surgeries. A different vantage point is depicted in *Please don't gayify!: an autoethnographic account of medicalised relationality for LGBTQI*+ *safe affirming medical health education and clinical practice* (Vicars and Deppeler), which narrates encounters with health professionals and medical training contexts to expose systemic inadequacies in LGBTQI+ inclusive healthcare.

*Toward promoting resilience of gender and sexually diverse youth in South African rural school ecologies* (Zhange and Mohangi) examines the experiences of LGBTQ+ youth in rural schools, demonstrating how teacher support and inclusive practices can foster resilience despite systemic heteronormativity and limited resources. *Building a model of navigational strategies for queer undergraduate students in STEM* (Voigt et al.) offers a cyclical model that captures how queer students evaluate environments, calculate risks, and enact strategies to resist marginalization in higher education. *A needs-assessment survey of the high school LGBTQ*+ *environment by a health science center interprofessional team* (Velasquez et al.) reports on the health and educational needs of LGBTQ+ youth in U.S. high schools and recommends targeted interventions to promote safer and more inclusive learning contexts.

In *Immigration, language education, & trauma: exploring the intersectionality of gay Dominican immigrant experiences*, González employs case study methodology to analyse the compounded trauma faced by gay Dominican men in New York City, illuminating the intersections of race, language, sexuality, and migration. *Unfolding the layers of LGBTQ*+ *identity, resilience, and multicultural perspectives in Vietnam* (Lam-Nguyen) offers a reflexive narrative that traces the challenges of growing up queer in Vietnam, focusing on bullying, cultural transition, and the importance of family and intercultural solidarity.

*Dressed like boys, hair trimmed, a nalla kutti otherwise”: construction of queer suicide in Indian online news media* (Jetubhai) critiques the sensationalist and stigmatizing representation of queer suicide in Indian journalism, calling for curricular reforms in media training. *Beyond the Iron Throne: exploring the representation of homosexuality in the series Game of Thrones* (Louis and Chithra) demonstrates the ambivalent portrayal of queer characters in a globally influential media franchise, demonstrating how representation oscillates between complexity and sensationalization. Finally, *Iconoclasm of normative structures: exploring queer ageing in “Kaathal: The core” by Jeo Baby* (Shree and Chithra) uses cinematic analysis to foreground queer aging and non-traditional kinship, presenting alternative imaginaries of belonging beyond heteronormative family structures.

## Final reflections

Researching sensitive topics is undeniably risky. As the sections of this editorial demonstrate, queer trauma is irrefutably complex and has required much thought, articulation, and revision in the production of this edited Research Topic. As a collective, we have advocated for Queer Trauma to be understood beyond the binaries of victimhood and discrimination. We recognize that researching and writing about queer lives means that often researchers, including those who have contributed to this edition, are laying themselves bare by declaring their own positionalities (Sikes, 2006). We believe that the risks are worth taking for the advancement of inclusion and social justice, and we are therefore grateful to all contributors.
